# Intrathoracic solitary fibrous tumor – an international multicenter study on clinical outcome and novel circulating biomarkers

**DOI:** 10.1038/s41598-017-12914-2

**Published:** 2017-10-02

**Authors:** Bahil Ghanim, Sebastian Hess, Pietro Bertoglio, Ali Celik, Aynur Bas, Felicitas Oberndorfer, Franca Melfi, Alfredo Mussi, Walter Klepetko, Christine Pirker, Walter Berger, Imrich Harmati, Attila Farkas, Hendrik Jan Ankersmit, Balazs Dome, Janos Fillinger, Clemens Aigner, Balazs Hegedus, Ferenc Renyi-Vamos, György Lang

**Affiliations:** 1 Division of Thoracic Surgery, Department of Surgery, Medical University of Vienna, Währinger Gürtel 18-20, 1090 Vienna, Austria; 2 Institute of Cancer Research, Medical University of Vienna, Borschkegasse 8a, 1090 Vienna, Austria; 3 Division of Thoracic Surgery, Department of Surgery, University Hospital of Pisa, Via Paradisa 2, 56124 Pisa, Italy; 4 Department of Thoracic Surgery, Gazi University School of Medicine, Besevler, 06500 Ankara, Turkey; 5 Department of Pathology, Medical University of Vienna, Währingergürtel 18-20, 1090 Vienna, Austria; 6 Robotic Multidisciplinary Center for Surgery-Robotic and Minimally Thoracic Surgery, University Hospital of Pisa, Via Paradisa 2, 56124 Pisa, Italy; 7 Department of Thoracic Surgery, National Institute of Oncology and Semmelweis University, Ráth György utca 7-9, H-1122 Budapest, Hungary; 8 Christian Doppler Laboratory for Cardiac and Thoracic Diagnosis and Regeneration, Medical University of Vienna, Währingergürtel 18-20, 1090 Vienna, Austria; 9 National Koranyi Institute of Pulmonology, Piheno út 1, H-1121 Budapest, Hungary; 10 Department of Pathology, National Institute of Oncology, Ráth György utca 7-9, H-1122 Budapest, Hungary; 11 Department of Thoracic Surgery, Ruhrlandklinik, University Duisburg-Essen, Tüschener Weg 40, Essen, D-45239 Germany; 12 MTA-SE Molecular Oncology Research Group, Hungarian Academy of Sciences - Semmelweis University, Üllői út 93, H-1091 Budapest, Hungary

## Abstract

Intrathoracic solitary fibrous tumor (SFT) is a rare disease. Radical resection is the standard of care. However, estimating prognosis and planning follow-up and treatment strategies remains challenging. Data were retrospectively collected by five international centers to explore outcome and biomarkers for predicting event-free-survival (EFS). 125 histological proven SFT patients (74 female; 59.2%; 104 benign; 83.2%) were analyzed. The one-, three-, five- and ten-year EFS after curative-intent surgery was 98%, 90%, 77% and 67%, respectively. Patients age (≥59 vs. <59 years hazard ratio (HR) 4.23, 95 confidence interval (CI) 1.56–11.47, p = 0.005), tumor-dignity (malignant vs. benign HR 6.98, CI 3.01–16.20, p <0.001), tumor-size (>10 cm vs. ≤10 cm HR 2.53, CI 1.10–5.83, p = 0.030), de Perrot staging (late vs. early HR 3.85, CI 1.65–8.98, p = 0.002) and resection margins (positive vs. negative HR 4.17, CI 1.15–15.17, p = 0,030) were associated with EFS. Furthermore, fibrinogen (elevated vs. normal HR 4.00, CI 1.49–10.72, p = 0.006) and the neutrophil–to-lymphocyte-ratio (NLR > 5 vs. < 5 HR 3.91, CI 1.40–10.89, p = 0.009) were prognostic after univariate analyses. After multivariate analyses tumor-dignity and fibrinogen remained as independent prognosticators. Besides validating the role of age, tumor-dignity, tumor-size, stage and resection margins, we identified for the first time inflammatory markers as prognosticators in SFT.

## Introduction

Intrathoracic solitary fibrous tumor (SFT) is a rare disease. In the past, the clinical management was complicated on the one hand by a complex and in part misleading terminology and on the other hand by the lack of diagnostic markers and histopathological characterization. However, progress in terms of modern pathology and genetic characterization have led to a more standardized nomenclature, improved biological understanding and thus more precise diagnosis and clinical management^1,2^.

SFT is currently defined as benign or malignant tumor of mesenchymal origin^3^. Furthermore, with regard to genomic alterations, the NAB2-STAT6 gene fusion was found to be a distinct genetic hallmark of this orphan disease^4^. NAB2-STAT6 gene fusion subtypes and the mutations in the promoter of TERT demonstrated prognostic significance in SFT^5,6^. Furthermore, STAT6 expression detected by immunohistochemistry in tumor tissue was found to be an additional useful marker to verify the diagnosis of intra- and extrathoracic SFT^7,8^.

Despite the recent advances in molecular and pathological SFT characterization, there are no consensus clinical guidelines considering treatment regimens and follow-up strategies. The current standard approach is curative-intent surgery aiming at complete resection of the tumor regardless of its dignity. In contrast to surgery, the role of conventional chemotherapy, radiotherapy and targeted therapy remains to be clarified^1^.

Predicting outcome of SFT patients is still challenging^9^. Up to 80% of all SFT are histologically classified as benign. Nevertheless, also benign tumors can recur and transform to the malignant variant. Overall, 15–20% of all SFT patients develop recurrence after initial treatment^1,10^.

Individual histopathological parameters demonstrated only limited prognostic power in SFT^11^. Accordingly, several scores that combine histological and clinical parameters were developed to improve the prediction of recurrence and survival^12–15^. However, most information is currently derived from pathological findings and no reliable non-invasive prognostic biomarker exists at the moment^16^.

Accordingly, a better risk stratification is urgently needed to estimate the clinical course and allocate patients to different follow-up and treatment strategies. Consequently, besides characterizing this rare disease comprehensively with regard to its clinical and pathological characteristics, we focused on analyzing the prognostic role of blood derived non-invasive routine markers in SFT.

Previously our study group – together with others - proved that circulating inflammatory biomarkers including C-reactive protein (CRP), fibrinogen and the neutrophil-to-lymphocyte ratio (NLR) are prognostic and – with regard to fibrinogen and CRP – predictive in the setting of multimodality therapy benefit for patients suffering from malignant pleural mesothelioma (MPM)^17–20^. Furthermore, we showed that an inflammatory status has a negative impact on outcome of patients receiving curative pulmonary metastasectomy for metastatic colorectal cancer^21^. Thus we hypothesized that inflammatory blood derived biomarkers might also harbor prognostic power in patients suffering from the other primary pleural tumor besides MPM - namely intrathoracic SFT.

## Results

### Clinical characteristics and postoperative morbidity and mortality

In summary, 74 female (59.2%) and 51 male (40.8%), histological proven SFT patients were analyzed. The mean age and standard deviation was 59 ± 12.2 years, ranging from 25 to 86 years. The most important clinical and pathological baseline characteristics of our study cohort are given in Table 1. Around half of the study population were never smokers (n = 56; 44.8%). Interestingly, 17 patients (13.6%) were suffering from other malignancies besides SFT.

**Table 1:** Patients characteristics of the whole study cohort (n = 125).

		number (%)
Gender	Female Male	74 (59.2%) 51 (40.8%)
Smoking status	Never smoker	56 (44.8%)
	Ever smoker	53 (42.4%)
	Missing data	16 (12.8%)
Recurrence of disease	No recurrence	111 (88.8%)
	Recurrence	14 (11.2%)
Surgery	VATS	34 (27.2%)
	Open	91 (72.8%)
Additional therapy	Systemic therapy	6 (4.8%)
	Radiotherapy	7 (5.6%)
	Chemo-Radiotherapy	2 (1.6%)
	Surgery alone	110 (88%)
Tumor dignity	Benign	104 (83.2%)
	Malignant	21 (16.8%)
Tumor size	≤10 cm	86 (68.8%)
	>10 cm	39 (31.2%)
Resection margins	Negative	112 (89.6%)
	Positive	9 (7.2%)
	Missing	4 (3.2%)
De Perrot Staging	0	49 (39.2%)
	1	38 (30.4%)
	2	14 (11.2%)
	3	19 (15.2%)
	4	1 (0.8%)
	Missing	4 (3.2%)
CD34	Positive	125 (100%)
	Negative	0
Bcl2	Positive	66 (52.8%)
	Negative	0
	Missing	59 (47.2%)
CD99	Positive	34 (27.2%)
	Negative	0
	Missing	91 (72.8%)
Median CRP (range) mg/dl (n = 62)		0.59 (0.2–35)
Median Fibrinogen (range) mg/dl (n = 79)		350 (200–1098)
Median NLR (range) (n = 111)		2.51 (0.93–16.38)

Abbreviations: video assisted thoracic surgery – VATS, C-Reactive Protein – CRP.

The majority of all patients was treated by open surgery (n = 91; 72.8%). Median hospital stay was 7 days (range 2–43 days) for all patients. Patients receiving video assisted thoracic surgery (VATS) (n = 32) or robotic surgery (n = 2) were summarized to the VATS group (n = 34; 27.2%). The VATS group had significantly shorter hospital stay when compared to patients treated by open surgery (median hospital stay VATS vs. open surgery: 5 vs. 7 days, Mann Whitney U test, p < 0.001). However, tumors treated by VATS were also significantly smaller (median tumor-size VATS vs. open surgery: 3.25 vs. 8.3 cm, Mann Whitney U test, p < 0.001) and always benign (number of malignant SFT treated by VATS vs. open surgery: 0 vs. 21, chi-square test: p = 0.002). Nevertheless, the proportion of R0 resection was comparable between the VATS and open surgery group (R0 resection in 31 VATS (93.9%) vs. 81 in open surgery patients (92%), Fisher’s exact test p = 1.000) indicating, that VATS is appropriate to achieve R0 resection in small and benign tumors.

In 15 (12%) patients postoperative complications occurred after primary resection (postoperative bleeding in seven patients, arrhythmia in two patients, neurological complications in two patients, wound infection in one patient, pneumonia in one patient, fluidopneumothorax in one patient and finally recurrent nerve palsy in another patient). Postoperative complication rate tended to be higher in the open surgery group (n = 14; 15.4% complication rate) when compared to the VATS group (n = 1; 2.9% complication rate, Fisher exact test p = 0.067, type of complication: recurrent nerve palsy). 30-day mortality was present in one patient (0.8%) who had postoperative bleeding and deceased despite all resuscitation attempts.

In summary, 14 patients (11.2%) experienced recurrence of disease and 16 patients (12.8%) died during the observation period. Recurrence was treated in 9 patients (64.3%) by surgery and in 5 patients (35.7%) by chemo- or radiotherapy.

In general, 6 patients (4.8%) were additionally treated by systemic therapy (chemo- and/or targeted therapy), 7 patients (5.6%) by radiotherapy and 2 patients (1.6%) by chemo- and radiotherapy besides radical resection of the primary tumor. The remaining 110 patients (88%) received curative-intent surgery as the single treatment.

### Pathological and immunohistochemical characterization

Most patients suffered from benign SFT (n = 104; 83.2%). Interestingly, malignant tumors were more frequently found in older than in younger patients (5 patients with malignant SFT younger than mean study population age of 59 years (23.8%) vs. 16 patients older than 59 years (76.2%), chi-square test p = 0.028). Furthermore, malignant tumors were significantly associated with a tumor-size bigger than 10 cm (12 malignant tumors > 10 cm (57.1%) vs. 9 smaller than 10 cm (42.9%), chi-square test p = 0.005).

Of note, most recurrent patients initially suffered from malignant SFT. Nevertheless, also benign tumors recurred despite microscopic radical R0 resection in 6 patients (recurrence rate 8 malignant (38.1%) vs. 6 benign (5.8%), Fisher exact test p < 0.001). Interestingly, recurrence of benign tumors was always limited to the chest (ipsilateral chest n = 4, both or contralateral chest n = 2). However, malignant SFT also recurred at extrathoracic sides (n = 3: brain, liver, and ilium bone).

De Perrot staging of our study cohort as well as the other major pathological characteristics are given in Table 1. Staging was summarized as early (de Perrot stages 0 and 1, n = 87; 69.6%) vs. late stage (de Perrot stages 2, 3, 4 and 5, n = 34; 27.2%). In 4 patients (3.2%) information about de Perrot staging was missing.

**Figure 1:**
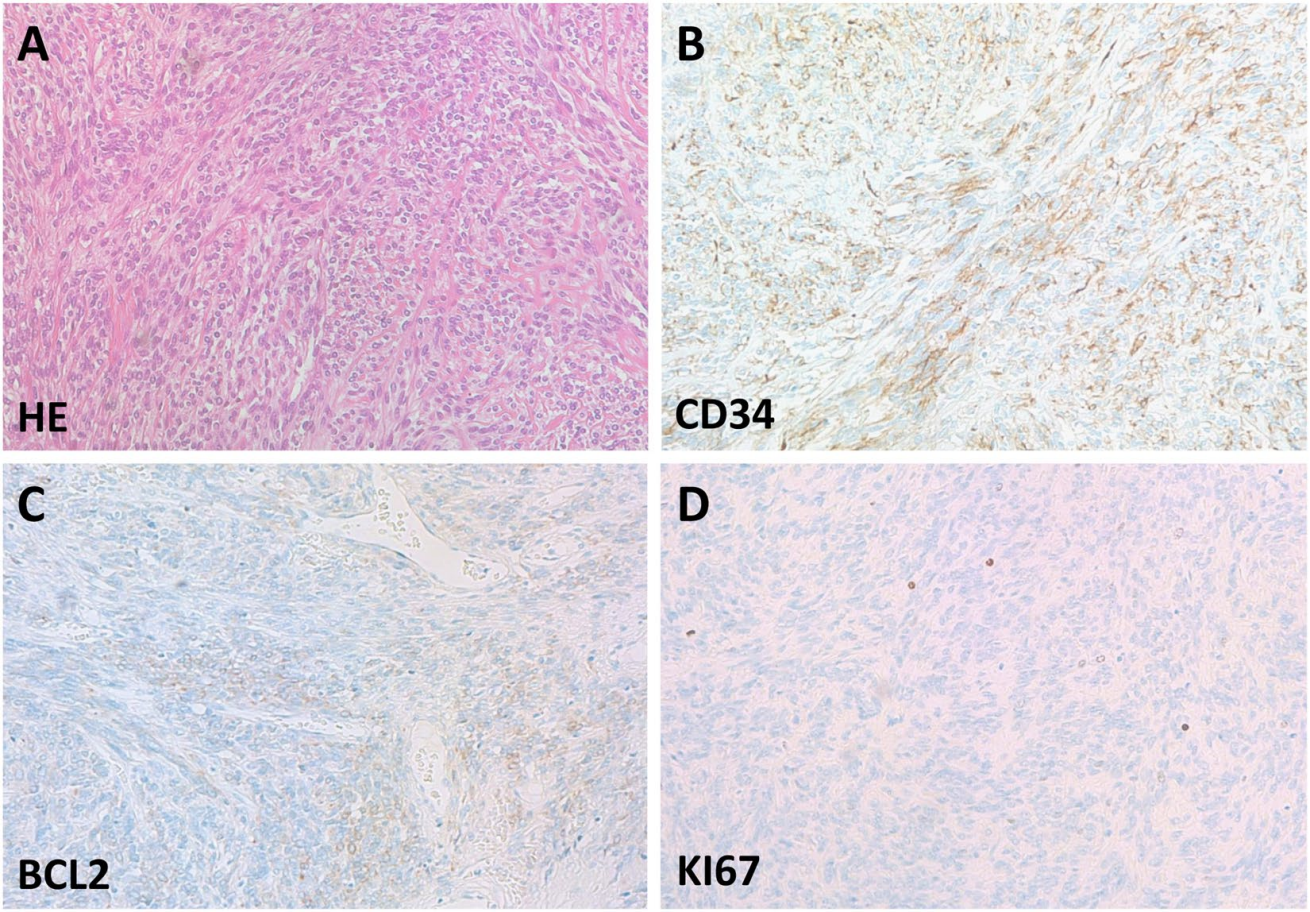
Histological features of solitary fibrous tumors. (**A**) The hematoxylin-eosin staining demonstrates relatively uniform tumor cells intimately intertwined between fibers. (**B**) Diffuse immunostaining of CD34 is a histological hallmark of the disease. (**C**) Focal labeling of BCL2 on the SFT tumor cells. (**D**) Sparse Ki67 positivity indicates the proliferating cells in the SFT tissue.

With regards to the immunohistochemical expression profile (Fig. 1), all patients initially analyzed during pathological routine workup were suffering from CD34 positive tumors (n = 114; 91.2% vs. 0 CD34 negative tumors). Additionally, all 11 patients without retrospective CD34 scoring were tested positive for this marker in staining performed for the current study. In general, the major diagnostic markers of SFT besides CD34 were expressed by all of the studied tumors (bcl2 positive cases n = 66; 52.8% vs. 0 bcl2 negative cases, CD99 positive cases n = 34; 27.2% vs. 0 CD99 negative cases). However, it has to be pointed out, that not all diagnostic markers were stained in every patient during routine workup. CD34 was the most frequently stained marker during routine pathological workup (91.2% of the study population) indicating that most cases were diagnosed by CD34 positivity and general histological, microscopic SFT features. Nevertheless, bcl2 and CD99 positivity was observed in all of the stained cases indicating that these markers are indeed useful to confirm SFT diagnosis. Of note, the very recent novel diagnostic marker of SFT, namely STAT6 expression^7,8^ was not performed in this retrospective patient cohort as the overwhelming majority of cases were diagnosed prior to the identification of this marker.

### Clinical and pathological characteristics of prognostic importance

Median EFS for all patients was not reached during the observation period reflecting the relative good outcome of SFT after curative–intent surgery. The 30-day EFS after resection was 99%, one-, three-, five- and ten-year EFS were 98%, 90%, 77% and 67% respectively.

From the clinical and pathological baseline characteristics patients age (as continuous characteristic, HR 1.049, CI 1.008–1.092, p = 0.019; dichotomized by mean age, ≥59 years vs. <59 years, HR 4.231, CI 1.561–11.465, p = 0.005), tumor-dignity (malignant vs. benign, HR 6.98, CI 3.01–16.20, p < 0.001), tumor-size (>10 cm vs. ≤ 10, HR 2.527, CI 1.096–5.825, p = 0.030), de Perrot staging (late vs. early de Perrot stage, HR 3.849, CI 1.651–8.975, p = 0.002) and surgical resection margins (positive vs. negative, HR 4.172, CI 1.147–15.169, p = 0.030) were found to be significantly associated with EFS after univariate survival analyses whereas gender and type of surgical approach (VATS vs. open surgery) did not influence the outcome after radical resection.

### Inflammation related circulating biomarkers in the SFT cohort

Median preoperative CRP level in our study population was 0.59 mg/dl (range 0.02–35 mg/dl). CRP was elevated (according to the clinical cut-off of 0.5 mg/dl) in 36 (28.8%) and normal in 26 patients (20.8%). However, in 63 patients (50.4%) preoperative CRP values were missing. Median fibrinogen of all patients was 350 mg/dl (range 200–1098 mg/dl). Fibrinogen levels were classified as elevated (according to the clinical cut-off of 390 mg/dl) in 33 patients (26.4%) and normal in 47 patients (37.6%). Fibrinogen levels were not available in 45 patients (36%). The median blood NLR was 2.51 (range 0.93–16.38). In 99 patients (79.2%) the NLR was lower than 5 and in 12 (9.6%) patients the NLR was higher than 5. In 14 patients (11.2%) either the neutrophil and/or the lymphocyte blood count was missing and thus the NLR could not be calculated.

### Association of inflammatory blood parameters with pathological characteristics

With regard to tumor-dignity, malignant SFT (non-significantly) tended to be associated with elevated fibrinogen (patients with malignant SFT having elevated vs. normal fibrinogen n = 11; 61.1% vs. 7; 35.5%, p = 0.052). Benign and malignant SFT patients did not differ significantly with regard to preoperative NLR or CRP values. Interestingly, patients with elevated CRP and high fibrinogen had significantly bigger tumors than patients with normal inflammatory parameters (median tumor-size elevated vs. normal CRP 10.15 vs. 4.95 cm, Mann Whitney U test p = 0.008; elevated vs. normal fibrinogen 11.0 vs. 6.5 cm, Mann Whitney U test p = 0.006). However, the difference between the high and the low NLR group with regard to tumor-size missed statistical significance (Mann Whitney U test p = 0.079). Furthermore, late stage of disease (late vs. early de Perrot stage) was found to be associated with elevated fibrinogen (late stage of disease elevated vs. normal fibrinogen n = 13; 61.9% vs. 8; 38.1%, chi-square test p = 0.031) but no such association was found for CRP (chi-square test p = 0.855) and the NLR (chi-square test p = 0.175).

### Inflammatory circulating biomarkers as prognostic parameters in SFT

**Figure 2:**
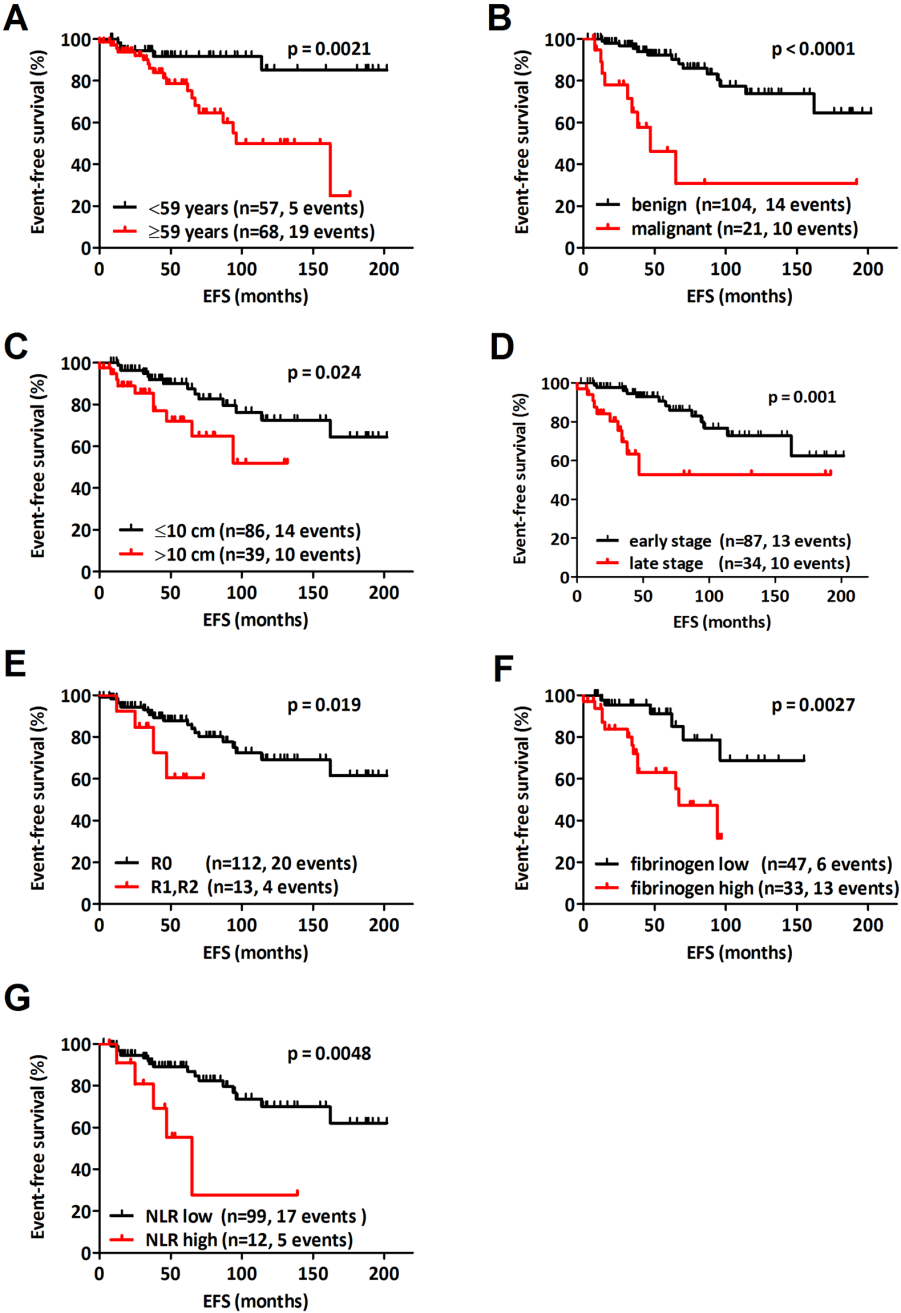
Kaplan-Meier graphs of the significant prognostic parameters. (**A**) Patients older than 59 years showed a significantly shorter event-free-survival (EFS) after curative-intent solitary fibrous tumor (SFT) surgery when compared to younger patients (n = 125, one-, three-, five- and ten-year 95 vs. 98; 86 vs. 94; 64 vs 92; and 50 vs. 85% respectively, log rank test p = 0.002). (**B**) Malignant SFT was characterized by significant shorter EFS when compared to benign SFT (n = 125, one-, three-, five- and ten-year EFS 85 vs. 100; 62 vs. 95; 44 vs. 92 and 29 vs. 74%, respectively, log rank test p < 0.001). (**C**) Patients with tumors bigger than 10 cm had the significant worse prognosis when compared to smaller tumors (n = 125, one-, three-, five- and ten-year EFS 92 vs. 100; 85 vs. 92; and 65 vs. 82%; 52 vs. 72%, log rank test p = 0.024). (**D**) The impact of the de Perrot staging system on EFS (n = 121, one-, three-, five- and ten-year EFS late vs. early de Perrot stage: 91 vs. 100; 69 vs. 96; 52 vs. 86 and 52 vs. 73%, respectively, log rank test p = 0.001). (**E**) Positive resection margins after curative-intent surgery were associated with shortened EFS when compared to free resection lines (n = 121, one-, three-, five- and ten-year EFS 89 vs. 98; 78 vs. 91; 52 vs. 80; and 52 vs. 69%, log rank test p = 0.019). (**F**) Circulating fibrinogen level had prognostic significance in SFT (n = 80, one-, three-, five- and ten-year EFS elevated vs. normal fibrinogen: 94 vs. 98; 72 vs. 95; 47 vs 78 and 32 vs. 69% respectively, p = 0.003). (**G**) The neutrophil-to-lymphocyte-ratio was prognostic in univariate EFS analysis (n = 111, one-, three-, five- and ten-year EFS NLR > 5 vs. < 5: 91 vs. 99; 81 vs. 90; 28 vs. 82 and 28 vs. 70% respectively, p = 0.005).

Using univariate Cox regression analyses, fibrinogen (elevated/ ≥ 390 mg/dl vs. normal fibrinogen, HR 4.001, CI 1.493–10.722, p = 0.006) and NLR ( > 5 vs. < 5 HR 3.906, CI 1.401–10.889, p = 0.009) but not CRP (elevated ≥ 0.5 mg/dl vs. normal CRP HR 3.185, CI 0.697–14.557, p = 0.135) were identified as prognostic blood derived biomarkers in SFT. The respective data are shown in Table 2 and Fig. 2, summarizing all clinical and pathological parameters of univariate statistical significance.

**Table 2:** Univariate prognostic characteristics.

Characteristic	Hazard ratio	95% confidence	p value
Age ≥ 59 vs. < 59 years	4.231	1.561–11.465	0.005
Dignity malignant vs. benign	6.981	3.008–16.204	<0.001
Tumor size > 10 cm vs. ≤ 10 cm	2.527	1.096–5.825	0.030
De Perrot Stage late vs. early	3.849	1.651–8.975	0.002
Resection margins positive vs. negative	4.172	1.147–15.169	0.030
Fibrinogen elevated vs. normal	4.001	1.493–10.722	0.006
NLR > 5 vs. < 5	3.906	1.401–10.889	0.009

Abbreviations: neutrophil-to-lymphocyte – NLR.

To further explore the prognostic role of inflammatory parameters in SFT, multivariate survival analyses were performed. Since there was a clear association between late de Perrot stage and malignant SFT, two Cox regression models including one of these parameters each were performed as illustrated in Table 3. In both multivariate models fibrinogen but not the NLR was an independent prognostic factor besides tumor-dignity.

**Table 3:** Multivariate survival analyses.

	Model 1 (n = 71)			Model 2 (n = 67)		
Number of events	17			16		
Number without event	54			51		
**Characteristic**	**HR**	**95% CI**	**p**	**HR**	**95% CI**	**p**
Age ≥ 59 vs. < 59 years	1.61	0.47–5.55	0.452	1.74	0.49–6.11	0.390
Dignity malignant vs. benign	4.66	1.22–17.80	0.024			
Tumor size > 10 cm vs. ≤ 10 cm	2.47	0.64–9.63	0.192	2.09	0.56–7.83	0.276
De Perrot Stage late vs. early				4.31	0.95–19.49	0.058
Resection margins positive vs. negative	2.99	0.45–19.99	0.258	2.98	0.46–19.09	0.250
Fibrinogen elevated vs. normal	4.55	1.20–17.17	0.026	4.44	1.11–17.69	0.035
NLR > 5 vs. < 5	2.50	0.49–12.88	0.274	4.11	0.71–23.76	0.114

Abbreviations: neutrophil-to-lymphocyte – NLR.

Model 1 multivariate cox regression including following characteristics: patient age, tumor dignity, tumor size, surgical resection margins, fibrinogen and the NLR opposed to.

Model 2 consisting of age, tumor size, de Perrot stage instead of tumor dignity, surgical resection margins, fibrinogen and the NLR.

## Discussion

In this international multicenter study the outcome of 125 histological proven intrathoracic SFT patients treated by curative-intent surgery was analyzed. Of note, this relatively large SFT study demonstrates for the first time independent prognostic quality of a blood-derived biomarker in patients suffering from this orphan disease.

The presented SFT cohort was representative in terms of the major clinical and pathological characteristics. The benign subtype accounted for 83.2% of our study population. This prevalence of benign SFT was close to the 80% found by Abu Arab^1^. Furthermore, as already described^22,23^, the malignant subtype was found to be associated with a larger tumor-size and worse outcome.

In contrast to lung cancer, only around half of all patients were smokers indicating that the pathogenesis of intrathoracic SFT is not as clearly related to tobacco consumption as known for lung cancer^24^. Similar to the literature, the prevalence of male and female SFT was almost equally distributed with a tendency towards more female than male cases (59.2% female). Also the mean age found in our study population was comparable to published data^14,22,23^.

In addition, age was also identified as prognostic characteristics in univariate survival analyses as shown before in one of the biggest SFT cohorts^25^. However, with regard to presence of other malignant diseases, we did not find a comparably high percentage of patients having other tumors besides SFT (13.6 vs. 27%) as published previously^22^. Interestingly, two patients had synchronous adenocarcinoma of the lung which was removed in both cases during primary SFT surgery by lobectomy. One patient died 5 years after surgery due to adenocarcinoma recurrence and the other patient is still alive without evidence of either disease 60 months after resection.

Importantly, our data suggest that VATS is a safe and efficient method to treat benign SFT of smaller size resulting in – compared to open surgery – a similar rate of tumor-free resection margins, shorter hospital stay and a lower complication rate. Furthermore, EFS did not differ significantly between the VATS and the open surgery group demonstrating, that the long- term oncological outcome is also not negatively influenced by applying minimal invasive surgery in SFT.

In addition, it has to be pointed out that two thirds of all recurrent SFT patients were eligible candidates for surgical treatment indicating that the follow-up should be managed by an interdisciplinary team including pulmonologists, radio-oncologists, oncologists but also thoracic surgeons. Thus, a lung parenchyma sparing but radical resection should be favored to keep the possibility for redo surgery in case of recurrence (only three out of 14 recurrent patients had extrathoracic recurrence).

Comparable to previous studies we verified the univariate prognostic value of the de Perrot staging in our study cohort^16,26^. Furthermore we proved in uni- and multivariate survival analyses, that tumor-dignity was prognostic independent from age, tumor-size, resection margins and the inflammatory parameters but was (as expected) strongly related to de Perrot stage as its power was completely lost when both pathological parameters were included in one multivariate model (data not shown). In addition, we showed, that tumor free resection margins and tumor-size impact significantly on postoperative outcome in univariate survival analyses as also shown before and again highlighting the central role of radical surgery in the management of SFT^27,28^.

For the first time, we demonstrated the independent prognostic value for preoperative fibrinogen in SFT receiving curative-intent surgery. Elevated fibrinogen showed a significant association with large tumor-size and advanced de Perrot stage. Furthermore, a – not significant but clear – tendency of higher fibrinogen in malignant SFT was detected. Most importantly, preoperative fibrinogen proved to be an independent prognostic marker predicting outcome after curative-intent surgery (independent from age, gender, resection margins, tumor-dignity and de Perrot stage). Taken together, these findings indicate that patients with elevated preoperative fibrinogen might suffer from a biological more aggressive SFT subtype translating to poor patient outcome. It suggests that distinct follow-up and treatment strategies after radical resection might be beneficial in this group of SFT patients.

Furthermore, these findings are in line with our previous experience regarding other thoracic malignancies^17,19,21^. However, in contrast to those studies mainly on mesothelioma, CRP was the only investigated inflammatory parameter that did not prove prognostic significance in SFT based on univariate survival analyses and thus was not further included in the multivariate testing. This might be at least in part explained by the relative low number of cases with documented CRP values (in the Italian cohort CRP was not measured at all during routine preoperative check-up resulting in n = 63; 50.4% cases with missing CRP). Furthermore, it has to be mentioned that the median overall survival as well as the median event free survival have both not been reached during our follow-up period (mean follow-up of 50 months) that might influence the statistical analysis for the prognostic value of CRP in our cohort. Indeed, patients with normal CRP before surgery had a tendency towards longer EFS especially in the long term follow-up (log rank test p = 0.115, CRP high vs. low: one-, three-, five- and ten-year survival: 94 vs. 95; 80 vs. 89; 46 vs. 89 and 31 vs. 89% respectively). This tendency might reach significance in a bigger cohort with more CRP values and/or longer follow-up period available. In addition, CRP values were measured by a different method in the Turkish cohort when compared to the rest of the study population resulting in 20 patients having CRP measurements performed in a different way.

Nevertheless, it has to be mentioned that patients with one or more than one characteristic missing (including CRP, fibrinogen, NLR, de Perrot stage, and resection margins; n = 85) did not differ significantly from the group of patients with all parameters available (n = 40; data not shown) with regard to age, sex, tumor dignity, de Perrot stage, tumor size and resection margins. Thus the group of patients with missing data was assumed to be representative for the whole study cohort. However, statistical power was indeed negatively influenced by the low number of patients in case of the prognostic value for CRP.

Furthermore, CRP was found to be significantly associated with larger tumor-size. Interestingly, the values of CRP and fibrinogen were strongly correlating with each other (Pearson´s correlation coefficient 0.676, p < 0.001) indicating, that both acute phase response proteins have a similar biological background (data not shown). However, the correlation between the NLR and the two acute phase response proteins was not that strong (Pearson´s correlation coefficient 0.262, p = 0.047 for CRP and NLR and Pearson´s correlation coefficient 0.253, p = 0.029 for fibrinogen and NLR) showing that the NLR might reflect another inflammatory axis in SFT than CRP and fibrinogen (data not shown).

Similar to our previous findings in malignant pleural mesothelioma, elevated fibrinogen was associated with late stage of disease^17^. These findings indicate that more advanced tumors induce enhanced systemic inflammatory response or alternatively produce higher amount of autocrine (inflammatory related) growth factors promoting angiogenesis and proliferation, thus leading to elevated systemic inflammatory marker production by the liver or bone marrow. This is also suggested by comparing median tumor-size (instead of de Perrot stage) directly between the high and the low fibrinogen group.

Nevertheless, it has to be mentioned, that all investigated inflammatory parameters including CRP, fibrinogen and the NLR are unfortunately also characterized by their low specificity since all markers are primarily used to detect and monitor systemic inflammation. However, all studied markers were measured before any surgical intervention in patients clinically judged to be fit for elective oncological surgery after exclusion of acute inflammatory disease. Furthermore, these markers have the advantage of being routine biomarkers which can be uniformly and at a low cost measured all over the world.

Inflammation plays a key role during development and progression of many malignancies. Consequently, the interaction between the immune system and the tumor is now also included in the recent hallmarks of cancer published by Hanahan and Weinberg^29^. The exact mechanisms that are underlying the prognostic power of systemic inflammatory parameters in cancer patients are currently not fully understood. However, it is well known, that cancer and inflammation share many common pathways regulating important characteristics of tumor survival and progression such as angiogenesis, migration and proliferation as reviewed by Balkwill *et al*.^30^ thus also offering novel therapeutic targets for already established anti-inflammatory compounds in the setting of modern oncology.

Finally, it has to be pointed out, that the modified immune response and escape from the immune system by the tumor is also a novel and promising therapy target for patients suffering from different malignancies as reviewed by Topalian *et al*.^31^.

Taken together, we here describe for the first time a prognostic role of non-invasive inflammatory markers in intrathoracic SFT. These inexpensive and widely available blood derived biomarkers might provide additional prognostic information after curative-intent surgery in this orphan disease. Furthermore our data indicate that an activated systemic inflammatory response is associated with a more aggressive SFT phenotype. Of note, our findings suggest that these markers should be explored in extrathoracic SFT patient cohorts as well. Furthermore, these inflammatory biomarkers should also be explored in the setting of monitoring disease progression or recurrence. Last but not least, the potential normalization of these inflammatory parameters after complete surgical tumor removal could also be investigated in a prospective setting.

Thus we concluded that complete SFT resection results in high disease control rates. VATS showed promising postoperative results in small and benign tumors. However, 11% of all patients suffered from recurrent disease after curative-intent surgery. Of note, even benign tumors recurred in 6 patients despite R0 resection. Thus a better risk stratification is required to design adequate follow-up strategies. Fibrinogen showed independent prognostic value and thus might aid in planning the personalized clinical management after resection. However, further validation of our findings in additional, preferentially prospectively studied SFT cohorts is needed to establish the aforementioned parameters in clinical routine.

## Patients and Methods

### Patients

Initially, 162 cases were identified as SFT patients at the five participating institutes. 14 patients were excluded as SFT was recurrent at first admission to the participating institute and information about the primary presentation was not available. Five patients were excluded due to the lack of curative-intent surgery. 12 patients were lost to follow-up. Six patients were excluded during the histological review that revealed two MPM, one neuroma, one synovial sarcoma, one inflammatory pseudo-tumor and one fibrosarcoma. Finally 125 patients were analyzed. Retrospective data collection was approved by the national or local ethics committees (Table 4) and the study was conducted according to the Helsinki Declaration and the guidelines for good scientific practice of the respective institutions. All 125 patients received curative-intent surgery between January 1999 and February 2016 and the diagnosis was proven by histology and immunohistochemistry at the participating centers by local specialized thoracic pathologists. Median follow-up after curative-intent surgery for all patients still alive during the observation period was 50 months (range 3–202 months) including only seven patients (5.6%) with follow-up less than 12 months. These seven patients had postoperative CT scan 3 months (n = 1) or 6 months (n = 5) after surgery available for the follow-up.

**Table 4:** Participating centers and ethical approvals.

Department	Ethic committee approval number	Number of patients
National Koranyi Institute, Budapest, Hungary	Hungarian Medical Research Council (52614–4/2013/EKU)	35
Division of Thoracic Surgery, University of Pisa, Italy	Bioethics Committee, University of Pisa (791/2015)	28
Division of Thoracic Surgery, Medical University of Vienna, Austria	Medical University of Vienna Ethic Committee (1671/2014)	24
Department of Thoracic Surgery, Gazi University School of Medicine, Ankara, Turkey	Gazi University Ethic Committee 129/2015	20
Department of Thoracic Surgery, National Institute of Oncology - Semmelweis University, Budapest, Hungary	Hungarian Medical Research Council (52614–4/2013/EKU)	18

Staging was performed as previously suggested by de Perrot *et al*. In brief, de Perrot stage 0 and 1 are defined by absence of histological signs of malignancy either with a pedunculated (stage 0) or sessile (stage 1) growth pattern. De Perrot stage 2 and 3 are characterized by histological signs of malignancy either pedunculated (stage 2) or sessile (stage 3). Finally, stage 4 is defined as metastatic disease^14^.

Malignancy was defined by the England criteria as published before taking the presence of the following histopathological characteristics into account: high mitotic activity, high cellularity, necrosis, cellular pleomorphism and hemorrhage^15^.

All inflammatory parameters were measured from peripheral venous blood sampled at day of admission or within seven days before first SFT surgery as part of preoperative routine check-up. The value closest to but before initial surgery was used for study purposes. CRP was measured as previously described by turbidimetric immunoassay^32^ in the same standardized way by all but not one center and the clinical cut-off (0.5 mg/dl) was used for dichotomizing to the elevated and normal CRP groups. CRP was measured by the nephelometric test only in the Turkish cohort. Fibrinogen values were determinate in all participating centers by the Claus method^33^ and considered as elevated when higher than 390 mg/dl. Finally, the NLR was calculated by dividing the blood neutrophil by the blood lymphocyte count. The NLR was considered high when bigger than 5 as published before for MPM by our group^19^.

The mean age (mean 59 ± 12.2 years) was used to dichotomize patients into the two age groups. With regard to tumor-size we employed the same cut-off (10 cm) as published before for intrathoracic SFT using the pathological report as source of information^23^.

### Statistical analyses

Metric data is always given as median and corresponding range if not otherwise indicated. Mann-Whitney U Test was utilized to compare the means of non-parametric distributed data. Unpaired t-test was used for parametric distributed data. Pearson’s chi-square test and Fisher’s exact test were performed as applicable to investigate the association between categorical factors. Event-free-survival (EFS) is reported in all survival analyses and was calculated as time between first surgery and death/recurrence or time from first surgery to last follow-up in patients still alive and without any evidence of disease. Survival analyses were performed by the Kaplan-Meier method and the log rank test. Furthermore, the Cox-regression was utilized to calculate the hazard ratios (HR) and corresponding confidence intervals (CI) for uni- and multivariate survival analyses. Multivariate survival analyses included the characteristics of univariate prognostic value. Two models were calculated since tumor-dignity and de Perrot stage were strongly correlating with each other. The two models consisted of 1) age (dichotomized by the mean age of 59 years), tumor-dignity (malignant vs. benign), tumor-size (>10 cm vs. ≤10 cm), resection margins (positive vs. negative), fibrinogen (high vs. low) and NLR (>5 vs. <5); and 2) age (dichotomized by the mean age of 59 years), de Perrot staging (late vs. early), tumor-size (>10 cm vs. ≤10 cm), resection margins (positive vs. negative), fibrinogen (high vs. low) and NLR (>5 vs. <5). All statistical analyses were calculated with SPSS Statistics 21 package (IBM®). *P* values are always given as two-sided and were considered statistically significant below 0.05.
